# Factors in the Development of Somatoform Disorders Among Children: A Case-Control Study

**DOI:** 10.7759/cureus.43238

**Published:** 2023-08-09

**Authors:** Hassan A Chaudhry, Chinwe C Okonkwo, Pugazhendi Inban, Tarsha A Intsiful, Uchenna E Ezenagu, Victor A Odoma, Sumit Kumar, Syeda Sarah Mahjabeen, Soumya Suvra Patra, Nishi M Modi, Aponinuola T Fewajesuyan, Sahar Firas Nabeel Makkiyah, Munira Abdefatah Ali, Aadil Khan

**Affiliations:** 1 Biological Sciences, Temple University, Philadelphia, USA; 2 Medicine, Medical University of Lublin, Lublin, POL; 3 Family Medicine, Caribbean Medical University School of Medicine, Willemstad, CUW; 4 General Medicine, Government Medical College, Chennai, IND; 5 Medicine, University of Ghana Medical Centre, Accra, GHA; 6 Medicine, Sumy State University, Sumy, UKR; 7 Cardiology/Oncology, Indiana University (IU) Health, Bloomington, USA; 8 Internal Medicine, Armed Forces Medical College, Pune, IND; 9 Pathology and Laboratory Medicine, Madinah Maternity and Children Hospital, Al-Madinah al-Munawwarah, SAU; 10 Pathology and Laboratory Medicine, Nandamuri Taraka Rama Rao University of Health Sciences (NTRUHS), Hyderabad, IND; 11 Internal Medicine, Calcutta National Medical College, Kolkata, IND; 12 Medicine, Government Medical College, Surat, IND; 13 Internal Medicine, Lviv National Medical University, Lviv, UKR; 14 Pathology, Baghdad University College of Medicine, Baghdad, IRQ; 15 Medicine, University of Illinois, Chicago, USA; 16 Internal Medicine, Lala Lajpat Rai (LLR) Hospital, Kanpur, IND

**Keywords:** intelligence quotient (iq), parental control, parental care, parental handling, somatoform disorders

## Abstract

Introduction

Somatoform disorders (SFDs) are a spectrum of diseases mainly manifesting with physical symptoms of no recognizable etiology. These disorders are believed to be primarily influenced and exacerbated by psychological factors. Given the connection between parental sociodemographics and psychological factors and SFDs, there is a pressing need to investigate this area further, particularly concerning parents and their children affected by somatoform disorders.

Aims and objectives

The aims and objectives of this study are as follows: i) study the determinants of SFDs, namely, parent handling of child, parent-child relationship, parenting with respect to attending to the needs of children, and intelligence quotient (IQ) of parents, and ii) compare host factors to the factors matched in control subjects.

Materials and methods

We adopted purposive sampling in our case-control study. The study sample was obtained from the psychiatry department of the Ganesh Shankar Vidyarthi Memorial (GSVM) Medical College, Kanpur, India, from September 2020 to December 2022, once a week, every Monday. Children manifesting SFD manifestations that are among the chief complaints were included in our study.

Results

A total of 115 cases were included in our study based on inclusion criteria. The study compared sociodemographic characteristics, intelligence quotient (IQ), parental characteristics, parental handling, parent-child relationships, and parenting practices between a patient group and a control group. The results showed no significant differences in sex, religion, occupation, domicile, and socioeconomic status in both control and patient groups. However, significant differences were found in parental characteristics, such as lower mean age, education, and IQ, in the patient group. This difference between the patient and control groups with respect to the parental handling questionnaire was statistically significant for the domain of "praise," "talk," "feel better," "comes to you," "unduly strict" items, "frequently reprimanded," "tried to control everything," creative activities, protectiveness, education, neglecting, objective punishment, housing, medical care, demanding, symbolic reward, loving, objective reward, food, parent-to-child communication, clothing, support, routine, recreation, social activities, rules, managing problem behavior, guidance for career, and security.

Conclusion

Parents were deficient in terms of years of education, occupational status, IQ, parental handling, parent-child relationship, and parenting with respect to the children's needs. These findings offer insights into the sociodemographic and psychological factors contributing to the patient group's condition.

## Introduction

The term somatoform disorder (SFD) originated from the Greek word "soma," which means body, and was first coined by Robert Spitzer. Somatoform disorders are a spectrum of diseases mainly manifesting with physical symptoms of no recognizable etiology. Somatoform disorders are predominately triggered and exacerbated by psychological factors [1]. There are six SFD subtypes, namely, somatization disorder, hypochondriasis, conversion disorder, body dysmorphic disorder, undifferentiated SFD, and SFD not otherwise specified, based on the Diagnostic and Statistical Manual of Mental Disorders, Fifth Edition, Text Revision (DSM-V-TR). However, the International Classification of Diseases, Tenth Revision (ICD-10) includes seven SFD subtypes, namely, somatization disorder, persistent panic disorder, hypochondriasis, dissociative disorder, undifferentiated SFD, somatoform autonomic dysfunction, and SFD unspecified. SFD in children is triggered by environmental, behavioral, psychological, and biological factors [2]. Research data on the essential domains of the etiological determinants of SFDs is not widely reported. The Neo-Freudian thinkers recognized environmental factors as more appealing than Freud's instinctual model, and they also observed that parent-child relationship is also an essential determinant of childhood psychiatric disorders [3-5].

The primary objectives of this study are twofold. Firstly, we aim to explore the determinants of SFDs in parents, focusing on aspects such as their handling of the child, the parent-child relationship, parenting practices regarding attending to the needs of children, and the intelligence quotient (IQ) of the parents. Secondly, we seek to compare these factors in the host group (parents of children with SFDs) to a control group to assess their significance in contributing to the condition.

## Materials and methods

Study design

We used purposive sampling, and the study sample was selected from the patients visiting the psychiatry department of the Ganesh Shankar Vidyarthi Memorial (GSVM) Medical College, Kanpur, India, from September 2020 to December 2022, once a week, every Monday. Children manifesting somatic symptoms were examined in detail.

Inclusion and exclusion criteria

The inclusion criteria include children (5-18 years old) diagnosed with SFDs based on DSM-V-TR [6]. All the children presenting with one or more somatic manifestations as chief complaints were screened after fulfilling the inclusion criteria, and each subject was assessed based on a detailed history, clinical examination, investigations, referrals, and the sociodemographic status of the family. The healthy children without psychiatric history (psychopathology measurement score of <10) were included in the control group. The control group was matched with the patient group in all available parameters, including sex, age, social status, education, and socioeconomic status. Consent was taken by using consent performa. Children with significant morbidity, psychiatric history, or IQ of <70 were excluded from the study. This included sociodemographic data, details of the history of present illness, past and family history, history of medical illness, personal history, and physical and mental status examination.

Data collection

The parental handling questionnaire scale was employed for the parents of the patients and controls for the assessment of parental handling of the child. It is a 14-item scale. It measures two parental handling variables, namely, "parental care" (10 items) and "parental control" (four items). This is a three-point scale where responses are recorded as "no," "sometimes," and "yes," to be scored as 0, 1, and 2, respectively [7]. Our study used the parent-child relationship scale (PCRS) test to assess the parent-child relationship between the patients and controls [8]. It contains 100 items, categorized into 10 dimensions: protective, neglecting, symbolic punishment, rejection, objective punishment, demanding, indifferent, symbolic reward, loving, and objective reward.

In a questionnaire for the assessment of parenting and the needs of children, a total of 18 items for the evaluation of parenting and the needs of children were identified, which are the following: food; clothes; housing; medical care; parent-to-child communication; child-to-parent communication; support: emotional and instrumental; routine: weekdays and holidays; education: study environment, monitoring, awareness of the level of performance, and effort by parents to improve performance; recreation: both indoors and outdoors; creative activities: availability and encouragement; social activities; rules/guidelines for behavior: beating, hitting, shouting, abusing, going out of home, borrowing things, pocket money, intake of substance, borrowing money, girlfriend/boyfriend, and mobile and fast food; moral and religious training: stealing, telling lies, morals and spirituals, and managing problem behavior; guidance on career; subject choice; security of the child; and equitable distribution: between siblings of same sex and different sex. On each item, after preliminary inquiry, the child and parents were to report whether the needs were met satisfactorily (0), mildly deficient (1), moderately deficient (2), or severely deficient (3). Clarifications were sought to arrive at a consistent scoring whenever doubts were expressed.

In the parental handling questionnaire, parents were asked to respond to the 14 questions of this scale with "no," "sometimes," or "yes," which was recorded on the scale as 0, 1, and 2. Separate scoring was done of the first 10 items of the scale for the assessment of "care" and the last "four" items in the evaluation of "control." Clarifications were sought to arrive at a consistent scoring whenever doubts were expressed.

In the parent-child relationship scale, patients were asked to rate 100 statements mentioned in the PCRS as to their own perception of their relationship with their father and mother on a five-point scale ranging from "always" to "very rarely" weighted 5, 4, 3, 2, and 1, on the scale points. The method of answering the statements was already explained to the child. It was ensured that no statement was left unanswered. Separate scoring was done for the father and mother. Clarifications were sought to arrive at a consistent scoring whenever doubts were expressed.

In the parenting assessment questionnaire, parenting, in terms of caring for the child's needs, was assessed with the help of a semi-structured questionnaire. The questionnaire was given jointly to the child and the available parent. Questions were regarding 18 significant needs of children. On each item, after preliminary inquiry, the child and parents were to report whether the needs were met satisfactorily (0), mildly deficient (1), moderately deficient (2), or severely deficient (3). Clarifications were sought to arrive at a consistent scoring whenever doubts were expressed. The parents of each child were assessed for IQ by Short Bhatia Battery Intelligence Test [9].

Data analysis

Statistical analysis was performed using appropriate methods, such as t-tests, chi-square (c^2^) tests, or analysis of variance (ANOVA), to compare the differences between the patient and control groups in terms of sociodemographic characteristics, parental characteristics, IQ, parental handling, parent-child relationship, and parenting practices. The significance level was set at p<0.05.

## Results

A total of 115 cases that fulfilled the selection criteria were studied. The sociodemographic characteristics of the sample are depicted in Table 1 and Table 2.

**Table 1:** Sample characteristics: sociodemographic variables. df, degree of freedom; c^2^, chi-square; N, number; NS, nonsignificant

Variables	Patient (n=75)		Control (n=40)		c^2^	df	p-value
	N	%	N	%			
Sex					0.051	1	NS
Female	54	72	28	70			
Male	21	28	12	30			
Religion					0.611	1	NS
Hindu	69	92	35	87.5			
Muslim	6	8	5	12.5			
Occupation					1.086	1	NS
Student	73	97.3	40	100			
Others	2	2.7	0	0			
Domicile					3.761	2	NS
Rural	46	61.3	17	42.5			
Semiurban	17	22.7	13	32.5			
Urban	12	16	10	25			
Socioeconomic status					3.783	2	NS
Upper middle	9	12	5	12.5			
Lower middle	51	68	25	62.5			
Lower	15	20	10	25			

**Table 2:** Sample characteristics: age and years of education. SD, standard deviation; n, number; df, degree of freedom; NS, nonsignificant

Variables	Patient (n=75)		Control (n=40)		t-value	df	p-value
	Mean	SD	Mean	SD			
Age in years	14.77	3.45	14.17	2.88	-0.002	113	NS
Years of education	7.39	1.98	8.82	1.31	1.60	113	NS

The mean age of the patient group was 14.44±3.45 years (range: 6-18 years), and that of the control group was 14.17±2.88 years (range: 8-18 years). The mean years of education in the patient group was 7.39±1.98 years (range: 3-14 years), and that of the control group was 8.82±1.31 years (range: 4-14 years). The sociodemographic characteristics of the parents of the patient and control groups are depicted in Tables 3-6.

**Table 3:** Comparison of parental characteristics of the patient and control groups: age and years of education of fathers. SD, standard deviation; df, degree of freedom; n, number; NS, nonsignificant

Variables	Patient (n=75)		Control (n=40)		t-value	df	p-value
	Mean	SD	Mean	SD			
Age in years	42.26	5.73	41.47	4.90	0.74	113	NS
Years of education	9.20	4.85	11.52	5.33	-2.36	113	0.02

**Table 4:** Comparison of parental characteristics of the patient and control groups: occupation of fathers. c^2^, chi-square; N, number; df, degree of freedom

Variables		Patient (n=75)		Control (n=40)		c^2^	df	p-value
		N	%	N	%			
Occupation	Unemployed	4	5.3	1	2.5	7.64	3	0.05
	Unskilled	12	15.9	6	15.0			
	Semiskilled	22	27.3	5	17.5			
	Skilled, clerk and shopkeeper	20	26.6	10	25.0			
	Semiprofessional	12	15.9	12	30.0			
	Professional	5	6.7	6	15.0			
	Total (115)	75		40				

**Table 5:** Comparison of parental characteristics of the patient and control groups: age and years of education of mothers. SD, standard deviation; NS, nonsignificant; df, degree of freedom; n, number.

Variables	Patient (n=75)		Control (n=40)		t-value	df	p-value
	Mean	SD	Mean	SD			
Age in years	38.64	5.54	38.07	4.81	0.54	113	NS
Years of education	8.30	3.98	10.02	4.74	-2.05	113	0.04

**Table 6:** Comparison of parental characteristics of the patient and control groups: occupation of mothers. c^2^, chi-square; df, degree of freedom; N, number

Variables		Patient (n=75)		Control (n=40)		c^2^	df	p-value
		N	%	N	%			
Occupation	Homemaker	58	77.9	28	70.0	7.95	3	0.04
	Unskilled	7	9.1	1	2.5			
	Semiskilled	5	6.5	1	2.5			
	Skilled, clerk and shopkeeper	2	2.6	3	7.5			
	Semiprofessional	2	2.6	4	10.0			
	Professional	1	1.3	3	7.5			
	Total (115)	75		40				

The mean age of mothers in the patient group was 38.64±5.54 years (range: 26-52 years), while in the control group, it was 38.07±4.81 years (range: 28-50). The p-value was not statistically significant (Table 3). The mean years of education of mothers in the patient group were 8.30±3.98 (1-18 years), while in the control group, it was 10.02±4.74 (1-20 years). This difference between the patient and control groups for years of education in mothers was statistically significant (Table 5). Around 80% of mothers in the patient group were homemakers. About 15% were engaged in unskilled or semiskilled occupations. A total of 2.6% of mothers in the patient group were engaged in skilled and semiprofessional occupations, while the remaining were in professional occupations. Homemakers and unskilled and semiskilled workers were more frequent in the patient group, while skilled, semiskilled, and professional mothers were more frequent in the control group. This difference between the patient and control groups with respect to the occupation of mothers was statistically significant (Table 6).

The IQ of the patients and parents is depicted in Table 5. The patient group had significantly lower IQ as compared to the control group. The mean IQ of the patient group was 90.87±17.01 (range: 73-128), and that of the control group was 112.38±5.99 (range: 73-122). This difference between the patient and control groups with respect to the IQ of the patient was statistically significant (Table 7).

**Table 7:** Comparison of the IQs of the subjects, mothers, and fathers of the patient and control groups. SD, standard deviation; df, degree of freedom; n, number; IQs, intelligence quotients

Variable	Patient (n=75)		Control (n=40)		t-value	df	p-value
	Mean	SD	Mean	SD			
Subjects	90.87	17.010	112.38	5.999	-7.731	113	0.00
Mother	86.40	8.900	91.55	6.271	-3.250	113	0.00
Father	93.99	9.606	94.48	10.495	-0.251	113	NS

The mean IQ of mothers of the patient group was lower than that of the control group. The mean IQ of mothers in the patient group was 86.40±8.90 (range: 70-114), compared to the mean IQ of 91.55±6.27 (range: 70-115) of the control group. This difference between the patient and control group with respect to the IQ of mothers was statistically significant (Table 7). This difference between the patient group and the control group with respect to the IQ of fathers was not statistically significant (Table 7).

The comparison of parental handling scores of the patient and control groups is depicted in Table 8. As compared to the control group, parents in the patient group praised their children less, provided less help, talked/spent time with children less, put less effort into making them feel better, provided a less conducive environment to make children come to them when distressed, and were unduly strict/lenient. This difference between the patient and control groups with respect to the parental handling questionnaire was statistically significant for the domain of praise, talk, feel better, comes to you, and unduly strict items.

**Table 8:** Comparison of the parental handling questionnaire (care) of the patient and control groups. NS, nonsignificant; df, degree of freedom; c^2^, chi-square; N, number

Variables	Score	Patient (n=75)		Control (n=40)		c^2^	df	p-value
		N	%	N	%			
Smile	0	19	25.7	8	20.0	1.53	2	NS
	1	41	54.4	20	50.0			
	2	15	19.9	12	30.0			
Praise	0	36	47.8	9	22.5	7.25	2	0.02
	1	23	30.6	17	42.5			
	2	16	21.6	14	35.0			
Help	0	11	14.6	4	10.0	0.51	2	NS
	1	38	50.9	21	52.5			
	2	26	34.5	15	37.5			
Talk	0	34	45.2	7	17.5	9.20	2	0.01
	1	22	29.6	20	50.0			
	2	19	25.2	13	32.5			
Feel better	0	37	49.5	10	25.0	8.17	2	0.01
	1	21	27.9	18	45.0			
	2	17	22.6	12	30.0			
Comes to you when distressed	0	30	39.9	8	20.0	10.69	2	0.00
	1	33	44.2	15	37.5			
	2	12	15.9	17	42.5			
Allowed to do things	0	38	50.9	18	45.0	0.89	2	NS
	1	25	33.2	16	40.0			
	2	12	15.9	6	15.0			
Unduly strict	0	17	22.9	15	37.5	8.12	2	0.01
	1	28	37.2	27	42.5			
	2	30	39.9	8	20.0			
Allowed to take decisions	0	28	37.2	10	25.0	3.67	2	NS
	1	35	46.9	18	45.0			
	2	12	15.9	12	30.0			
Can take care of themselves	0	15	19.9	7	17.5	0.12	2	NS
	1	32	42.5	18	45.0			
	2	28	37.6	15	37.5			

A comparison of the parental handling questionnaire for the "control" patient and control group is depicted in Table 9. This difference between the patient group and the control group with respect to parental handling for "control" was statistically significant for domains of "frequently reprimanded" and "tried to control everything."

**Table 9:** Comparison of the parental handling questionnaire (control) of the patient and control groups. df, degree of freedom; c^2^, chi-square; N, number; NS, nonsignificant

Variables	Score	Patient (n=75)		Control (n=40)		c^2^	df	p-value
		N	%	N	%			
Frequently reprimanded	0	10	13.3	8	20.0	7.79	2	0.02
	1	26	34.9	22	55.0			
	2	39	51.8	10	25.0			
Protection from being reprimanded	0	37	49.5	16	40.0	2.18	2	NS
	1	27	35.9	20	50.0			
	2	11	14.6	4	10.0			
Get angry	0	10	13.3	7	17.5	2.86	2	NS
	1	46	61.5	18	45.0			
	2	19	25.2	15	37.5			
Try to control everything	0	10	13.3	9	22.5	6.47	2	0.03
	1	22	29.5	18	45.0			
	2	43	57.2	13	32.5			

A comparison of the parent-child relationship scale of "fathers" in the patient and control groups is depicted in Table 10. A total of 10 characteristics of parenting, namely, protective, neglecting, symbolic punishment, rejection, objective punishment, demanding, indifferent, symbolic reward, loving, and objective reward, were assessed with the parent-child relationship scale (PCRS). When a comparison was made between the patient group and the control group on PCRS scores of fathers, it was observed that fathers in the patient group had "lower mean scores" concerning items "protectiveness," "loving," "symbolic reward," and "objective rewards." Fathers had "higher mean scores" with respect to items "neglecting," "objective punishment," and "demanding." This difference between the patient and control groups with respect to protectiveness, neglecting, objective punishment, demanding, symbolic reward, loving, and objective reward was statistically significant (Table 10).

**Table 10:** Comparison of fathers of the patient and control groups on the parent-child relationship scale. SD, standard deviation; df, degree of freedom; NS, nonsignificant; n, number

Variable	Patient (n=75)		Control (n=40)		t-value	df	p-value
	Mean	SD	Mean	SD			
Protective	31.17	3.164	33.15	3.847	-2.956	113	0.00
Neglecting	25.24	3.962	20.88	3.722	5.744	113	0.00
Symbolic punishment	37.40	4.230	36.55	4.095	1.038	113	NS
Rejection	25.32	3.264	24.22	7.381	1.101	113	NS
Objective punishment	34.35	3.592	30.95	6.579	3.587	113	0.00
Demanding	35.65	3.482	33.15	5.772	2.900	113	0.00
Indifferent	24.27	3.843	23.55	4.607	0.888	113	NS
Symbolic reward	23.51	3.793	27.02	8.313	-3.115	113	0.00
Loving	23.23	2.989	27.70	7.286	-4.647	113	0.00
Objective reward	17.13	2.854	22.30	3.480	-8.555	113	0.00

A comparison of the parent-child relationship scale of "mothers" in the patient and control groups is depicted in Table 11. When a comparison was made between the patient group and the control group on PCRS scores of mothers, it was observed that mothers in the patient group had "lower mean scores" with respect to items "neglecting," "symbolic reward," "loving," and "objective reward." Mothers had "higher mean scores" with respect to items "protectiveness," "symbolic punishment," "rejection," "objective punishment," "demanding," and "indifference." They were more protective, used to give more symbolic and objective punishment, showed more rejection, and were more demanding and indifferent. This difference between the patient group and the control group with respect to protective, neglecting, symbolic punishment, rejection, objective punishment, demanding, indifferent, symbolic reward, loving, and objective reward was statistically significant (Table 11).

**Table 11:** Comparison of mothers of the patient and control groups on parent-child relationship scale. SD, standard deviation; df, degree of freedom; n, number

Variable	Patient (n=75)		Control (n=40)		t-value	df	p-value
	Mean	SD	Mean	SD			
Protective	33.21	3.550	24.45	6.508	9.359	113	0.00
Neglecting	25.04	4.018	35.52	4.006	-13.341	113	0.00
Symbolic punishment	35.08	3.392	20.70	4.513	19.244	113	0.00
Rejection	24.85	2.893	20.22	2.626	8.431	113	0.00
Objective punishment	36.12	3.226	28.55	7.182	7.793	113	0.00
Demanding	34.88	3.590	32.08	6.700	2.928	113	0.00
Indifferent	23.69	3.308	19.92	5.201	4.738	113	0.00
Symbolic reward	22.88	3.605	32.88	6.107	-11.040	113	0.00
Loving	22.95	3.031	30.58	7.702	-7.570	113	0.00
Objective reward	17.28	3.532	28.62	7.344	-11.197	113	0.00

A comparison of parenting in the patient and control groups on the parenting assessment questionnaire is depicted in Table 12. The patient and control groups were assessed on a semi-structured interview schedule for parenting concerning caring for children's needs. It was observed that the mean scores for parenting were higher (indicating various levels of deficiencies) in the patient group as compared to the control group with respect to items "food," "clothing," "housing," "medical care," "parent-to-child communication," "support," "routine," "education," "social activities," "rules," "managing problem behavior," "guidance for career," and "security." The lower mean score was observed for "recreation."

**Table 12:** Comparison of the patient and control groups on parenting assessment questionnaire. df, degree of freedom; NS, nonsignificant; SD, standard deviation; n, number

Variable	Patients (n=75)		Control (n=40)		t-value	df	p-value
	Mean	SD	Mean	SD			
Food	9.07	4.005	7.68	1.207	2.143	113	0.03
Clothing	7.88	2.711	5.68	2.411	4.312	113	0.00
Housing	17.13	2.591	11.30	2.174	12.136	113	0.00
Medical care	7.31	1.335	4.38	1.192	11.630	113	0.00
Parent-to-child communication	11.13	0.811	5.20	1.043	33.757	113	0.00
Child-to-parent communication	6.72	4.422	5.48	1.132	1.747	113	NS
Support	5.73	1.483	4.00	1.281	6.251	113	0.00
Routine	10.19	1.270	3.40	1.194	27.855	113	0.00
Education	14.33	1.223	6.02	1.121	35.702	113	0.00
Recreation	3.23	1.321	6.88	1.884	-12.111	113	0.00
Creative activities	3.16	1.151	5.88	1.937	-9.428	113	0.00
Social activities	7.41	2.308	5.68	1.927	4.065	113	0.00
Rules	38.05	4.815	26.75	3.894	12.776	113	0.00
Moral training	1.05	0.695	1.22	0.530	-1.363	113	NS
Managing problem behavior	3.16	0.698	1.25	0.494	15.360	113	0.00
Guidance for career	3.47	0.553	1.28	0.506	20.828	113	0.00
Security	1.65	0.762	1.38	0.586	2.013	113	0.04
Equitable distribution	2.51	1.614	2.30	1.244	0.705	113	NS

This difference between the patient and control groups for food, clothing, housing, medical care, parent-to-child communication, support, routine, education, recreation, creative activities, social activities, rules, managing problem behavior, and guidance for career and security was statistically significant (Table 12).

## Discussion

The sample in the present investigation comprised 75 patients and 40 healthy controls recruited through stringent selection criteria. When the parents of the patients and controls were compared, it was observed that the patient's parents had lesser years of education. It was further observed that in the patient group, more fathers were unemployed, unskilled, and semiskilled as compared to fathers in the control group. The latter was more in semiprofessional and professional jobs. Similar findings were observed concerning mothers. They had a greater frequency of homemakers and unskilled and semiskilled workers, while in the control group, more mothers had semiprofessional and professional jobs. In addition to these findings, a significant difference was found in the intelligence quotient (IQ) of mothers. The mothers of the patient group had lower IQs as compared to the mothers of the controls. These findings suggest that parents, especially mothers of the patients, were disadvantaged in terms of less education and having low levels of occupation and intelligence.

Parent handling of children is considered an important etiological factor for somatoform disorders [10,11]. Parental handling was assessed regarding two dimensions, that is, "care" and "control." In the patient group, parental care needed to have more frequent praise, talking to the child, making the child feel better when they are upset, the child approaching the parent in distress, and the parent being unduly strict/lenient. Similarly, it was observed that parental control was also deficient as parents in the patient group more frequently reprimanded their children and tried more regularly to control everything that the child did [12].

There needs to be more literature on parental handling in the country. Malhotra et al. observed that low care for younger children, high control for older children, inadequate care, high control for males, rural background, and high socioeconomic status were associated with psychiatric morbidity in children [13]. The findings of the present study also highlight the importance of low care and high control in the genesis of somatoform disorders. However, the present study did not compare parental handling in different sociodemographic subgroups, so the two studies cannot be compared. Besides, it is essential to mention that the study by Malhotra [14] was carried out almost a quarter of a century ago. Considering the rapid socioeconomic changes in the country and the significant challenges posed to parenting highlighted by Freud and Strachey, there is a need for systematic work in parenting [5].

A parent-child relationship is an essential determinant of child behavior, both healthy and unhealthy. It has been reported that mother-child relationship scores are better in normal children than father-child relationship scores in all dimensions of parent-child relationship scales, except object reward [10]. In the present study, parents, especially mothers, are deficient in many ways, and this has a definite relationship with somatoform psychopathology. It may be mentioned that in India, most males either stay outdoors or are engaged in full-time jobs and therefore are unavailable most of the time. Mothers have to take significant responsibility for caring for and handling the child's various needs, as they are nonworking and available at home [15].

Further assessment of children in relation to parents was done by parent-child relationship scale on 10 domains: protectiveness, neglecting, symbolic punishment, rejection, objective punishment, demanding, indifferent, symbolic reward, loving, and objective reward. It was observed that the father-child relationship in the patient group was deficient in seven domains, namely, neglecting, objective punishment, demanding, indifferent, symbolic reward, loving, and objective reward. Likewise, the mother-child relationship was also deficient in terms of being more protective, less neglecting, used to giving more symbolic and objective punishment, more rejective, more demanding, more indifferent, less loving, and used to providing less reward, both symbolic and objective [16-18].

Parenting in terms of the need of children was assessed on 18 domains ranging from basic needs, emotional needs, needs relating to education, and others by means of a semi-structured questionnaire. These findings suggest that multiple factors related to the patient's parents (e.g., parental handling of the child, parenting with respect to attending to the needs of children, IQ of parents, and history of psychiatric illness), parent-child relationship, and psychosocial stress in the recent past (last one year) have a role to play in the etiology of somatoform disorders in children. Many of these factors seem to be interrelated; for example, the lower occupational status of parents may be related to a deficient parent-child relationship, poor handling of the child, and care for the need of the child, especially concerning activity scheduling and monitoring of the child's educational and other needs [19,20].

Our study has limitations. One of the limitations of our study is low sample size, which limited the generalizability of the findings to a larger population of individuals with somatoform disorders. A larger and more diverse sample would provide a more representative picture of the disorder. Another limitation is selection bias. There might be a potential for selection bias in the study, as participants have been recruited from specific clinical settings. This could introduce a bias and limit the external validity of the findings. Our study might have used self-report measures or subjective assessments to gather data on symptoms and diagnostic criteria. While these instruments are commonly used, they can be prone to recall bias or subjective interpretation, potentially impacting the accuracy and reliability of the findings. Our study is cross-sectional in nature, providing a snapshot of the participants' experiences at a specific point in time. Longitudinal studies that follow individuals over an extended period would provide more insight into the natural course and progression of somatoform disorders. Another limitation is diagnostic challenges. Somatoform disorders can be complex to diagnose due to overlapping symptoms with other medical conditions. The study may have faced challenges in accurately diagnosing participants, which could impact the validity of the results.

## Conclusions

Our study revealed that parents in the patient group were deficient in terms of years of education, occupational status, IQ, parental handling (five domains of "parental care," viz., praise, talk, feel better, come to you when distressed, and unduly strict/lenient; two domains of "parental control," viz., frequently reprimanded and gets angry), parent-child relationship, and parenting with respect to attending the need of children. These findings suggest that children with the studied condition may experience less positive parenting, lower IQ scores, and deficiencies in various aspects of parenting compared to their healthy counterparts. These results highlight the importance of considering these factors in understanding and addressing the needs of children with the studied condition and their families. Further research is needed to explore these associations and their potential implications for intervention and support strategies.
